# Incidence and Reproduction Numbers of Pertussis: Estimates from Serological and Social Contact Data in Five European Countries

**DOI:** 10.1371/journal.pmed.1000291

**Published:** 2010-06-22

**Authors:** Mirjam Kretzschmar, Peter F. M. Teunis, Richard G. Pebody

**Affiliations:** 1 Julius Centre for Health Sciences and Primary Care, University Medical Centre Utrecht, Utrecht, The Netherlands; 2 Centre for Infectious Disease Control, RIVM, Bilthoven, The Netherlands; 3 Hubert Department of Global Health, Rollins School of Public Health, Emory University, Atlanta, Georgia, United States of America; 4 Health Protection Agency, London, United Kingdom; Harvard School of Public Health, United States of America

## Abstract

**Background:**

Despite large-scale vaccination programmes, pertussis has remained endemic in all European countries and has been on the rise in many countries in the last decade. One of the reasons that have been discussed for the failure of vaccination to eliminate the disease is continued circulation of the pathogen *Bordetella pertussis* by mostly asymptomatic and mild infections in adolescents and adults. To understand the impact of asymptomatic and undiagnosed infection on the transmission dynamics of pertussis we analysed serological data from five European countries in combination with information about social contact patterns from five of those countries to estimate incidence and reproduction numbers.

**Methods and Findings:**

We compared two different methods for estimating incidence from individual data on IgG pertussis toxin (PT) titres. One method combines the cross-sectional surveys of titres with longitudinal information about the distribution of amplitude and decay rate of titres in a back-calculation approach. The second method uses age-dependent contact matrices and cross-sectional surveys of IgG PT titres to estimate a next generation matrix for pertussis transmission among age groups. The next generation approach allows for computation of basic reproduction numbers for five European countries. Our main findings are that the seroincidence of infections as estimated with the first method in all countries lies between 1% and 6% per annum with a peak in the adolescent age groups and a second lower peak in young adults. The incidence of infections as estimated by the second method lies slightly lower with ranges between 1% and 4% per annum. There is a remarkably good agreement of the results obtained with the two methods. The basic reproduction numbers are similar across countries at around 5.5.

**Conclusions:**

Vaccination with currently used vaccines cannot prevent continued circulation and reinfection with pertussis, but has shifted the bulk of infections to adolescents and adults. If a vaccine conferring lifelong protection against clinical and subclinical infection were available pertussis could be eliminated. Currently, continuing circulation of the pathogen at a subclinical level provides a refuge for the pathogen in which it can evolve and adjust to infect vaccinated populations.

*Please see later in the article for the Editors' Summary*

## Abstract 2

Analyses of serological and social contact data from several European countries by Miriam Kretzschmar and colleagues show that vaccination against pertussis has shifted the burden of infection from children to adolescents and adults.

## Editors' Summary

**Background:**

Pertussis—whooping cough—is a highly infectious respiratory tract infection caused by *Bordetella pertussis* bacteria. The first symptoms of pertussis, which is spread by sneezing or coughing, are similar to those of a common cold. Then, after 1–2 weeks, severe outbursts of coughing develop that end with a characteristic “whoop.” Whooping cough is most common in infants and young children although adolescents and adults can develop a mild, often undiagnosed version of the disease. By contrast, more than half of children who develop pertussis before they are a year old need hospitalization. Complications of pertussis include pneumonia, a brain condition called encephalopathy and, in rare cases, death. Treatment with antibiotics can shorten the illness and can stop it spreading to other people. Whooping cough can be prevented by vaccination—an injection of inactive *B. pertussis* that “primes” the immune system so that it can deal quickly and effectively with any subsequent infections. Globally, more than 80% of children are now vaccinated against pertussis in the first few months of life.

**Why Was This Study Done?:**

Widespread pertussis vaccination since the 1950s has greatly reduced the incidence (the number of new cases in a population) of whooping cough. However, the disease is still present in all countries and its incidence is increasing again in many Western countries. One possible reason for vaccination failing to eliminate whooping cough might be *B. pertussis* transmission through mild infections in adolescents and adults. Because the protection provided by vaccination wanes over time, people who were vaccinated as infants can develop mild pertussis as adolescents or adults and unknowingly infect unvaccinated babies. Currently, pertussis vaccination involves several injections in the first few months of life and a preschool booster but some countries have recently introduced booster doses of pertussis vaccine for adolescents and adults to try to improve pertussis control, even though the transmission dynamics of pertussis in adults and adolescents are poorly understood. In this study, the researchers estimate the incidence and basic reproduction number (the expected number of new cases of a disease caused by an infected individual) in five European countries to discover more about the transmission dynamics of pertussis.

**What Did the Researchers Do and Find?:**

The researchers used two new statistical methods to estimate pertussis incidence from data collected in Finland, Germany, Italy, The Netherlands, and the UK in the mid-1990s (before the introduction of adolescent booster doses) on the levels of antibodies to pertussis in the blood of individuals (serological data). The first method used information on the decay rate of pertussis antibodies to estimate infection rates from these serological data. The annual incidence of pertussis infections estimated this way ranged from 1% in the UK to 6% in Italy and Germany with a peak of infections in adolescents and a second, weaker peak in young adults. The second method combined the serological data with information on daily social contacts in the populations of the same countries collected by the POLYMOD project (a study designed to strengthen public-health decision making in Europe). The annual incidence of infections estimated this way ranged between 1% and 4%, again with a peak of infection in adolescents. The basic reproduction number of pertussis infection estimated using the second method was about 5.5 in all five countries.

**What Do These Findings Mean?:**

Although the accuracy of the estimates reported here may be affected by the accuracy of the data entered into the calculations, these findings suggest that the incidences of pertussis in Finland, Germany, Italy, The Netherlands, and the UK are higher than the incidences obtained from national surveillance systems. They provide an indication, therefore, of the scale of underreporting and/or underdiagnosis of pertussis in these countries. In addition, they indicate that widespread vaccination with current vaccine schedules has shifted the bulk of pertussis infections to adolescents and adults who rarely develop severe pertussis. Thus, the researchers suggest, pertussis could be eliminated with moderate effort if a vaccine, or vaccine schedule, could be developed that provided life-long protection against pertussis. Finally and importantly, the two new methods described in this paper can now be used to find out whether the introduction of adolescent boosters has actually reduced the incidence of pertussis.

**Additional Information:**

Please access these Web sites via the online version of this summary at http://dx.doi.org/10.1371/journal.pmed.1000291.

- The US Centers for Disease Control and Prevention has a feature on pertussis (whooping cough) and provides detailed information on pertussis vaccination (in English and Spanish)
- KidsHealth, a resource provided by the Nemours Children's Health System, has information for parents on pertussis (in English and Spanish)
- The UK Health Protection Agency provides information about pertussis and pertussis vaccination
- The National Institute of Public Health and the Environment (RIVM) of the Netherlands provides information about pertussis (in Dutch)
- Information about whooping cough is available on the UK National Health Service Choices website
- Information about the surveillance of pertussis and other vaccine preventable diseases can be found on the European surveillance network EUVAC.NET website
- Links to additional resources are provided by MedlinePlus (in English and Spanish)
- Information on POLYMOD is available

## Introduction

There has been a long-standing discussion in the public health community about how mass vaccination impacts the circulation of *B. pertussis* in a population and how it reduces transmission [1],[2]. Over the last decade pertussis has reemerged in many countries where large-scale vaccination programmes have been in place since the middle of the last century. After a period during which infections with the bacterial pathogen *B. pertussis* seemed to be under control, pertussis incidence is now reportedly rising in many Western countries, even those with stable and high vaccination coverage. Several reasons have been put forward to explain these trends: a surveillance artifact due to the introduction of new diagnostic methods or a true epidemiological observation representing, for example, waning vaccine-induced protection. With respect to the latter explanation it is now known that subclinical infections in adolescent and adult populations may play a major role in the persistent circulation of *B. pertussis* in highly vaccinated populations [3]. Immunity after natural infection and vaccine-induced immunity both wane after some years and reinfection of previously infected or of vaccinated individuals have both been observed [4]. Prior immunization, as well as older age, usually results in a milder clinical presentation of the infection, such that pertussis infections in adults are often not recognized or diagnosed. Nevertheless, asymptomatically infected persons can be a source of new infection and can pose a threat to children who are too young to be vaccinated, and are therefore susceptible to potentially severe pertussis infection [5].

What is the most effective vaccination strategy to protect those who might suffer the most severe complications following infection with *B. pertussis*? Several mathematical modelling studies have been conducted to assess the effectiveness of various vaccination strategies on pertussis incidence [6]–[9]. All of those studies suffered from a lack of quantitative input information about the impact of subclinical infections on the transmission dynamics of *B. pertussis* and the difficulty in interpreting pertussis notification data. Nevertheless, following recommendations from such modelling analyses, adolescent or adult booster vaccination has been introduced in several countries. The so-called ‘cocooning strategy’ is also under discussion, an approach that consists of vaccinating household members of newborn infants [7]. An accurate assessment of the potential effectiveness of these vaccination strategies rests on a firm knowledge of the incidence of infection in older age groups and on quantitative estimates of the transmission potential of infected persons in these groups. Notification data are a weak source of information in that respect, because of the mostly asymptomatic/mildly symptomatic nature of pertussis in these older-age groups [10],[11]. Therefore other methods to estimate pertussis incidence have been developed using seroepidemiological approaches [12]. These approaches have used high titres of anti-pertussis toxin as a marker of recent infection [13]. A quantitative description of the time course of serum antibody titres following infection (seroconversion) allows translation of the titres in a cross-sectional sample into times since infection. The statistical methods first developed to analyse such data have since been extended and applied to other pathogens [14],[15]. For pertussis these studies have produced estimates in line with other epidemiological studies [16]–[18].

In a parallel development, the importance of a sound knowledge of contact patterns leading to infection was recognized in the context of mathematical modelling of infections spread by close contact [19],[20]. Therefore, social contact data were collected in several European countries in the years 2005/2006, enabling age-dependent mixing patterns to be described for eight European countries [21].

In this article, we bring these two branches of research together by combining the statistical methods for estimating seroincidence and the methods for estimating reproduction numbers of serological data and contact mixing matrices. We apply these methods to previously published standardized pertussis serological data from five European countries (Finland, Germany, Italy, The Netherlands, and United Kingdom) as collected during the mid-1990s prior to the introduction of adolescent booster doses [13]. We also use age-dependent contact data from the same countries, as collected during the POLYMOD project (a European Union funded project on “Improving Public Health Policy in Europe through the Modelling and Economic Evaluation of Interventions for the Control of Infectious Diseases”) [21]. We show that two different statistical methods result in comparable estimates for the incidence of pertussis infections in those countries and report on estimates for the basic reproduction number for pertussis infection.

## Methods

### Serological Data, Immunization Scheme, and Coverages

During the European Sero Epidemiology Network (ESEN) project serological data were collected in seven European countries for a number of infectious diseases, including pertussis. Standardization methods were developed for comparison of antibody titres among the countries such that the serological status of different populations could be compared. For pertussis the measurement of IgG pertussis toxin (PT) antibodies was standardized [22] and standardized measurements of the distribution of IgG PT titres were available for representative population samples from six different countries (Finland, Germany, Italy, The Netherlands, England and Wales, and France) across the entire age range. We decided to use the standardized IgG PT titres from the first part of the project, because the standardization was well established, the results are published, and at the time of the serosurveys undertaken in the mid-1990s, pertussis booster vaccination was not yet implemented in the majority of countries (Table 1). We do not report on the results for France, because for France there were no social contact data available.

**Table 1:** Vaccination schedules at time of serosurveys [13].

Country	Year of Serosurvey	Primary Course (mo)	Booster (mo)	Coverage in Period 1990–1999 (Percent)	Vaccine
**Finland**	1996	3, 4, 5	24	99	Whole cell
**Germany**	1995	3, 4, 5	12–15	85	Acellular since 1995
**Italy**	1996	3, 5, 7	No	53	Acellular since 1995
**The Netherlands**	1995	3, 4, 5	11	97	Whole cell
**UK**	1996	2, 3, 4	No	92	Whole cell

We utilised the previously published ESEN pertussis dataset [13] with individual records of IgG PT titre value by age, sex, and country. As there seemed to be no difference between males and females in the distribution of titre values, we did not further distinguish by gender. In addition to the serological data, we used information about the age distribution of the population in the different countries obtained from national population censuses as in [21].

### Social Contact Data

As described in more detail in [21], cross-sectional surveys of contact patterns were conducted in Belgium (BE), Germany (DE), Finland (FI), Great Britain excluding Northern Ireland (GB), Italy (IT), Luxembourg (LU), The Netherlands (NL), and Poland (PL). In all countries samples representative for the general population were aimed for with an oversampling of children of 0–5 y of age. Data collection was organized for each country separately and slightly different methodologies were employed: in six countries random digit phone dialling was used for the recruitment of participants, in two countries recruitment was performed face-to-face (random walking route) either as a separate study (Poland) or as a part of a multitheme survey (Germany). Participants in The Netherlands and Finland were recruited via population registers. The surveys were performed between May 2005 and September 2006. In The Netherlands the survey was part of a larger serological study and the sample provided for this analysis was smaller than for other countries. Participants received a self-administered questionnaire in the form of a diary and were asked to fill in each contact made during the course of one day. A contact was defined either as a two-way conversation in close proximity or as a conversation including physical contact like shaking hands or kissing. Among others, information about the age or age range of the contact person was collected. Since the study covered all age groups, two or three different versions of the questionnaire were used: in all countries but Germany, a questionnaire for children (0–14 y), adolescents (15–18 y), and adults (19+ y); in Germany only two versions of the questionnaire were used: for children or young adolescents (0–14 y) and for older adolescents or adults (15+ y). Questionnaires for young children were filled in by parents or guardians. Informed consent was obtained prior to the distribution of the questionnaire. From these questionnaires, data matrices can be derived that describe the frequencies of contacts between different age groups of a population. For the purposes here, we used contact matrices that describe the frequencies of contacts in populations subdivided into 15 age classes 0–4 y, 5–9 y, 10–14 y, …, 70+ y.

### Method for Estimating Incidence

In [12] and [15] a method for estimating incidence from serological measurements in a population is described. The method uses information about the serum antibody response to infection, peak levels, and decay rates of antibody titres, to back-calculate times since infection in a cross-sectional serum sample. This method can be simplified by assuming that new infections occur following a time homogeneous Poisson process. The marginal distribution of cross-sectional titres can then be described as a function of the known serum antibody response parameters and the unknown Poisson rate of infections (seroconversions). For pertussis, longitudinal data about the kinetics of IgG PT titres after infection are available from a study reported in [23]. Statistical models were fitted to these longitudinal data in [4],[24]. Both amplitude and decay rate of titres are highly variable among individuals, but their distributions can be well described on the population level. Estimates for these parameters can then be used to estimate the seroconversion rate for an observed cross-sectional IgG PT titre distribution. The underlying assumption is that the population from which the cross-sectional sample is taken is in endemic equilibrium. Using the parameter estimates obtained in [4], we estimated a rate parameter for incidence of new infections per 5-y age group from each of the five cross-sectional datasets from the ESEN study. In a sensitivity analysis we checked how the uncertainty of these estimates was related to the sample sizes in different age groups.

### Method for Estimating Basic Reproduction Number from Survey Data and Serological Data

In [20] a method was introduced to estimate the basic reproduction number (*R*_0_) from an age-mixing matrix in combination with serological data. As before, the method assumes that the population is in endemic equilibrium. It then applies the concept of the next generation matrix [25] to estimate the force of infection by age in an iterative procedure. In the original model, an underlying assumption was that infection implies lifelong immunity. For the purposes of this paper we modified the method and applied it with a different interpretation. Let *S*(*a*) denote the fraction of seronegative persons by age, and λ(*a*) the age-dependent rate of seroconversion. Furthermore, we denote by 1/γ the time that an individual remains seropositive after infection. Then the age-dependent fraction of seronegatives can be described by the differential equation
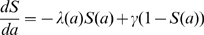
(1)with initial value 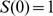. We use IgG PT titre values as a proxy for recent infection rather than a correlate of protection.

For classifying individuals as seronegative or seropositive, respectively, we used the cumulative density function
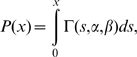
(2)where Γ(*s*,α,β) denotes the probability density of a Gamma distribution with shape parameter α and scale parameter β. The values for α and β were chosen such (α = 7.3 and β = 11.1) that at a cut-off value of 94 U/ml the sensitivity and specificity agree with values found for diagnostic testing by [26]. *P*(*x*) is then the probability that an individual with IgG PT titre *x* is classified as seropositive. The uncertainty in estimating the fraction seropositives per age group was assessed using bootstrapping methods on the basis of binomial distributions.

The value of the parameter 1/γ was estimated at 1.1 y on the basis of earlier estimates for the decay of antibody titres after infection. Combining these decay rates with the probability function in Equation 2 led to an estimate for the distribution of the time that an individual will be diagnosed as seropositive after infection. Then, the force of infection λ(*a*) was estimated with a maximum likelihood procedure that included an iterative process of determining the next generation matrix (see Text S1). Here the mixing between age classes was based on the symmetrized mixing matrices from the POLYMOD surveys for five countries (Finland, Germany, Italy, The Netherlands, UK). We did the analysis for the matrices on the basis of all contacts and the matrices on the basis of only those contacts that included physical contact. We compared the goodness of fit for both types of matrices using the Bayes Information Criterion (BIC). This is a statistical criterion for model selection based on the likelihood function with a penalty for the number of free parameters. From the next generation matrix, estimates for the basic reproduction number could then be computed as dominant eigenvalues. We conducted sensitivity analyses to study the impact of the assumptions about α, β, and γ on the estimates for *R*_0_ and the force of infection (unpublished results). Incidence per annum (*t* = 1) was then computed from the estimated fractions of seronegatives per age group and the force of infection by age as
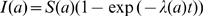
(3)By embedding the iteration procedure for obtaining the next generation matrix into a Markov chain Monte Carlo (MCMC) algorithm we computed uncertainty bounds (95% credible intervals) around the estimates for *R*_0_ and the incidence.

### Sensitivity Analysis Using Hypothetical Contact Matrices

To determine how strongly the incidence estimates that are based on the contact matrices depended on the specific form of the matrices, we computed estimates on the basis of two hypothetical contact matrices. One was a contact matrix describing homogeneous mixing between all age classes, i.e., all matrix elements were chosen as equal. The other hypothetical matrix was based on the matrix of all conversational contacts from the POLYMOD surveys, where we reduced the impact of assortative mixing within age classes by multiplication of the diagonal and first subdiagonal elements by factors 0.2 and 0.5, respectively. The rationale behind the second choice was that possibly contacts in households, which are often between parents and their children, might be of more importance for pertussis transmission than school or work contacts, which are strongly assortative between age groups.

All analyses were performed with Mathematica 7.0.

## Results

### Seroincidence of Pertussis in Five European Countries

**Figure 1:**
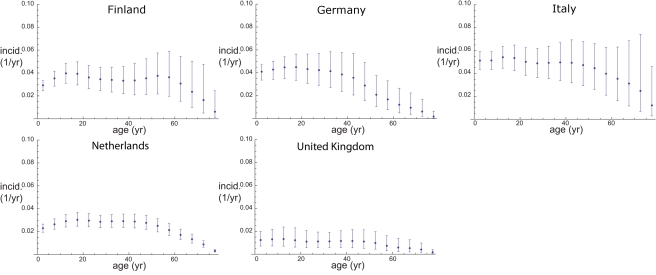
Seroincidence of pertussis in five European countries. Maximum likelihood estimates and (likelihood-based) 95% confidence intervals for the infection incidence as a function of age (5-y age classes).

Estimating incidences on the basis of longitudinal antibody decay data from The Netherlands and cross-sectional serological data from five ESEN countries resulted in the incidence estimates shown in Figure 1. Overall the annual incidence varies between around 1% in the UK up to around 6% in Italy and Germany. The distribution over age classes is comparable across countries with a peak in the adolescent age classes and a less pronounced peak in younger adults. The uncertainty in these incidence estimates varies among age groups and countries (compare The Netherlands with Finland and Italy, for example). These differences are mainly caused by different numbers of sera in each category.

### Estimates of the Force Of Infection and Basic Reproduction Number for Five European Countries

**Figure 2:**
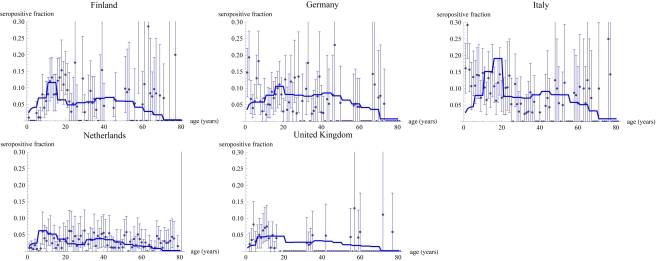
Seropositive fraction per age class from serological data using a standard diagnostic test (dots) and fitted model (solid line). The error bars represent 95% bootstrapping confidence intervals.

**Figure 3:**
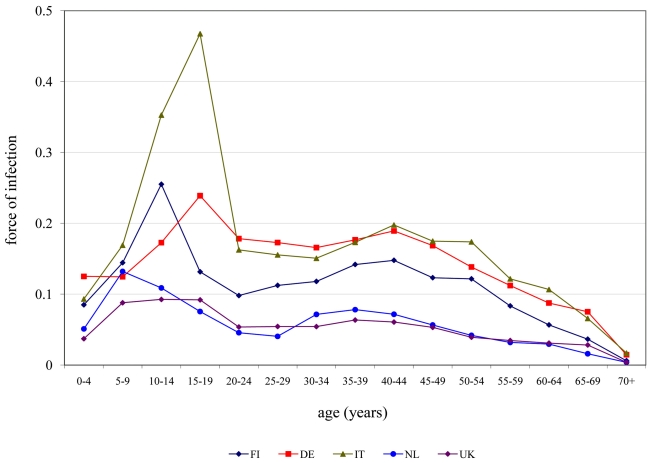
Estimates for the age-dependent force of infection (risk per year of becoming infected) for five European countries. DE, Germany; FI, Finland; IT, Italy; NL, The Netherlands.

Using the estimation method on the basis of the next generation matrix approach for the five countries for which both serological and contact data were available, we estimated the fraction per age class that is seropositive using a diagnostic test with a cut-off of 94 U/ml (Figure 2). Furthermore we estimated the age-dependent force of infection (Figure 3). Here The Netherlands and the UK have the lowest overall force of infection, and Italy and Germany the highest, whereas Finland takes an intermediate position. The basic reproduction number in an unvaccinated population can be computed as the dominant eigenvalue from the estimated next-generation matrices for each of the five countries (Table 2). The resulting values between 5 and 6 are similar across all five countries. When conducting the same analysis on the basis of contact matrices that included only physical contact, the estimates for the basic reproduction numbers remain virtually unchanged. The analysis using physical contact matrices does predict a higher value of the transmission parameter to account for the lower numbers of contacts available for transmission (unpublished data). A comparison of goodness of fit of the model with all contacts versus the model with only physical contacts shows differing outcomes per country (Table 3). While for Italy, Germany, and the UK the physical contact matrices give a slightly better fit, in Finland and The Netherlands the matrices with all contacts provide a better model.

**Table 2:** Estimates for the basic reproduction number (*R*_0_) based on the matrices for all contacts, only physical contacts, and two different hypothetical matrices.

Country	*R*_0_ All Contacts Matrix (95% CI)	*R*_0_ Physical Contacts Matrix (95% CI)	*R*_0_ Homogeneous Mixing Matrix (95% CI)	*R*_0_ All Contacts Matrix with Reduced Diagonals (95% CI)
**Finland**	5.5 (5.44–5.57)	5.5 (5.45–5.60)	5.7 (5.60–5.71)	5.4 (5.38–5.51)
**Germany**	5.6 (5.51–5.63)	5.5 (5.47–5.59)	5.8 (5.69–5.81)	5.7 (5.59–5.74)
**Italy**	5.9 (5.82–6.01)	5.9 (5.82–6.01)	5.8 (5.75–5.89)	5.8 (5.75–5.90)
**The Netherlands**	5.3 (5.26–5.35)	5.4 (5.30–5.42)	5.4 (5.34–5.42)	5.2 (5.18–5.26)
**UK**	5.4 (5.27–5.40)	5.2 (5.13–5.27)	5.5 (5.39–5.53)	5.3 (5.23–5.37)

Uncertainty estimates obtained by a Markov chain Monte Carlo (MCMC) algorithm (95% credible intervals [CIs]) in brackets.

**Table 3:** BIC values for model fit with four different contact matrices.

Country	BIC All Contacts Matrix	BIC Physical Contacts Matrix	BIC Homogeneous Mixing Matrix	BIC All Contacts Matrix with Reduced Diagonals
**Finland**	295.44	361.13	337.31	331.89
**Germany**	343.45	343.17	353.34	368.09
**Italy**	464.79	449.74	351.08	439.82
**The Netherlands**	402.32	497.69	347.92	345.02
**UK**	196.21	162.06	186.81	187.14

Two are empirical contact matrices from the POLYMOD study [21] and two are hypothetical contact matrices. The empirical matrices describe all conversational contacts and only physical contacts, respectively. As hypothetical contact matrices we chose a homogeneous mixing matrix and a matrix on the basis of the all-contacts matrix, with reduced diagonal and subdiagonal elements. A lower BIC value indicates a better model fit.

### Comparison of Incidence from Both Estimation Methods

**Figure 4:**
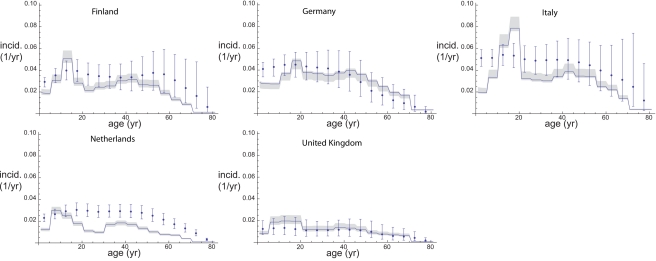
Comparison of incidence estimates from both estimation methods. Dots, seroincidence with error estimates; solid lines, incidence of infections that test positively with diagnostic tests with 95% credible intervals (shaded).

For the second estimation method incidence estimates can be computed from the age-dependent fraction of seronegatives and the force of infection. For the incidence computed in that way the age distribution differs somewhat from the seroincidences as estimated with the first method (Figure 4). The age distribution is more pronounced than for the seroincidence estimates with a strong peak in the adolescent age classes, especially in those countries with a high overall force of infection. In countries with a lower force of infection (The Netherlands, UK) the peak seems to lie in somewhat younger age classes than in the countries with a higher overall force of infection, maybe reflecting the faster loss of immunity after vaccination as compared with natural infection in young children. Overall, incidence estimates obtained with the second method are lower than those obtained with the first method.

### Hypothetical Contact Matrices

Sensitivity analysis using hypothetical contact matrices shows that the age distribution in incidence is strongly determined by the age-dependent mixing patterns. Homogeneous mixing predicts flat incidence profiles, while mixing with reduced assortative mixing produces incidence estimates that closely resemble those obtained by the back-calculation method (unpublished results). Comparing the goodness of fit of estimates on the basis of those matrices does not lead to clear conclusions about the optimal contact patterns. It is reassuring that the estimates of the reproduction number *R*_0_ are robust with respect to the details of the mixing patterns (Table 2).

## Discussion

We estimated the incidence of infection with *B. pertussis* during the 1990s from serological data for five European countries using two novel statistical modelling methods. One method uses information about the decay rate of antibodies against PT to estimate infection rates from IgG PT levels in cross-sectional sera. The other method uses information about age-dependent contact patterns and fractions of seropositive persons by age to derive an estimate for the force of infection. The two methods both lead to high incidence levels overall and for all countries, but there is also a clear difference between the estimates. We found the incidence levels were between 1% and 6% for all five countries for the first method and between 1% and 4% for the second method. Furthermore, although there was variation in incidence by country, the basic reproduction numbers were remarkably similar across countries, and for contact matrices based on all contacts versus only physical contacts. The incidences estimated here differ strongly from incidences found in national surveillance systems [11], clearly illustrating large underreporting rates and differences between countries in reporting rates.

The two estimation methods are based on very different ideas and approaches. The first method assumes that infections occur according to a time homogeneous Poisson process, whose rate can be estimated from cross-sectional serological data using information about amplitude and decay rate of the host immune response. The latter has to be estimated independently from longitudinal studies on antibody kinetics after infection. These methods have also been applied successfully to infections with other pathogens such as *Campylobacter* and *Salmonella* [15]. The method assumes that existing antibody levels do not confer any protection against renewed seroconversion, but considers dynamics of antibodies as a continuous process of antibody boosting and decay. Nothing is said about the incidence of symptomatic infections. The second method is based on the notion of a dynamic transmission model, where transmission is age dependent and mixing among age groups occurs according to an age mixing matrix. In this method, the population is subdivided into seropositive and seronegative individuals and an infection event moves an individual from the latter to the former. While the definition of seropositive can be chosen arbitrarily, here we used properties from a diagnostic test to calibrate the model, meaning that those found seropositive in the estimation procedure would be diagnosed as seropositive with this test. In other words, this second method does not consider all boosting events as infections, but only those that move an individual from being seronegative to seropositive. Therefore the second method leads to somewhat lower incidence estimates than the first method. Both methods rely on the assumption that the transmission dynamic process is at equilibrium. While the first method is more direct and based only on serological data, the second method considers immunity to infection and provides the possibility to estimate the force of infection and basic reproduction numbers.

Both methods showed a peak in incidence in adolescent age groups, a second lower peak in the young adult age groups, and a decreasing incidence at older ages. However, there are some differences between the incidence estimates obtained with the two methods. The incidence estimates using the contact matrix data had more pronounced peaks in the adolescent age groups compared to the incidence estimates that were derived only from the serological information. Comparing the explanatory power of using all contacts versus only physical contacts to estimate the force of infection led to interesting differences between countries. In countries with lower vaccination coverage and a higher peak age of transmission, physical contacts provided a slightly better proxy for describing transmission dynamics. In countries with high vaccination coverage and lower ages of peak transmission (with exception of the UK), the matrix with all conversational contacts provided a better fit. This might indicate that subclinical infections are transmitted through varying types of contacts, while clinical and severe infections require closer contact to be transmitted. The overall observed incidence levels presented here were comparable to the high titre prevalence estimates seen in the previous ESEN study [13] and were in line with earlier results based on a true back-calculation approach [12].

Comparing the performance of the POLYMOD contact matrices with two hypothetical contact matrices shows that the overall contacts patterns obtained by the POLYMOD surveys might not be optimally suited to describe pertussis transmission dynamics. The reasons for the suboptimal fit could be that pertussis transmission mainly occurs within households, where the assortative age mixing is less pronounced, or that duration and proximity of contacts have to be weighted by their duration, frequency, or proximity [27]. Possibly, the distribution of contacts among different settings, like households, schools, and workplace, has to be taken into account [28]. Our combined approach of incidence estimation methods opens up the possibility of investigating which types of contact matrices are best suited to describe observed seroepidemiological data and might therefore be able to give valuable hints about the main transmission routes and settings for specific pathogens.

Our estimates for the basic reproduction number are lower than those previously published [29]. However, earlier estimates were based on the assumption that natural infection provides lifelong protective immunity against future infections. Although the basic reproduction numbers estimated here apply to unvaccinated populations, we expect that the reproduction rates in vaccinated populations are not much different, because vaccination hardly seems to prevent repeated subclinical infections, even though it protects against symptomatic infection. The reproduction numbers were very similar across countries, and the observed small differences were consistent with the differences in vaccination schedules and coverages. Italy and Germany had somewhat higher reproduction numbers than the remaining countries and also displayed higher incidence estimates.

The values for the basic reproduction number of pertussis estimated here, lead us to conclude that pertussis is less transmissible than other childhood infections such as measles and rubella. Estimates of the *R*_0_ for measles are very high at around 20 [30] or more [31], for rubella the estimates are at around 7 [32], but both infections have a shorter infectious period than pertussis [33]. That means that an infected individual with pertussis produces fewer secondary infections during the infectious period on average than previously thought, which is promising for intervention. Waning immunity accounts for the difficulty in eliminating pertussis. Infected individuals find sufficient numbers of susceptible individuals around them to keep circulation going. This implies that elimination of pertussis will only be possible if a vaccine can be developed that also confers long-lasting protection against subclinical infections. The continued circulation of *B. pertussis* through mild and asymptomatic infections provides opportunities for the pathogen to evolve into strains that have increased virulence in unimmunized hosts, and current vaccines may not provide complete protection against such new evolved strains [34].

A potential limitation of our study is that contact patterns for children in the POLYMOD study were assessed by their parents [21]. Furthermore, respondents only gave information about the contacts made during a single day, so time trends in contact patterns cannot be taken into account. Finally, we only used information about the numbers of contacts and their distribution across age classes, and did not include information about the duration of contacts or the frequency of contact with the same person. We did, however, examine possible differences between all conversational contact and only physical contact, but did not find any effect of using only contacts that included some physical contact on our results. The data on social contacts were collected around 10 y after the serological data from the ESEN study. We assumed here that age-dependent contact patterns remained stable over that time period. Possible empirical support for this assumption comes from comparing the contact matrix in [20] with the contact matrices in [21]. There is a good agreement even if there is a time span of almost 20 y between the respective data collections.

Another limitation of our study is that vaccination with various types of pertussis vaccines might result in increases in titres of IgG PT; in this analysis we did not take the effects of vaccination on IgG PT titres into account. Although some pertussis vaccines hardly affect IgG PT titre levels, others can result in a large increase in IgG PT titre [13]. However, we did not see any vaccine-induced seroconversions in the longitudinal study (which included vaccinated and unvaccinated children). At the time the ESEN serological data were collected, vaccination in all participating ESEN countries was limited to the first 2 y of life [13]. Thus vaccination could have interfered with IgG PT levels in the youngest age group in some countries, and results in this age-group should be interpreted cautiously. Some high titres attributed to natural infection may have resulted from vaccination thereby leading to an overestimation of the incidence in the youngest age groups. However this would not be the case for older age-groups. Also, there were differences in the efficacy of the vaccines used in different countries at that time, which might partly explain differences in the force of infection in young children. Future investigation will probe how to translate seroincidence as estimated here into conclusions about burden of disease or incidence of notified infections.

Finally, the assumption of an endemic equilibrium that is made in both methods might not be supported by the data from the ESEN study. For many countries, an increase in pertussis incidence began in the 1990s, and epidemic peaks in incidence have been observed at regular intervals in some countries [35]. However, the ESEN study seems to have been conducted just before the major increase in incidence began. Also, recent infections that are characterized by high IgG-PT titres are less influenced by long term time trends, and therefore the estimation methods have an intrinsic robustness concerning time trends. However, as new cross-sectional serological studies are becoming available, we are planning to extend our methods to also account for the effects of changes in vaccination schedules and time trends.

It is known that low IgG PT titres are a correlate of susceptibility for symptomatic pertussis disease [36], but it is not known what proportion of all transmission events estimated here actually lead to symptomatic disease and notification. More clinical information about the relationship between transmission, infection, and symptoms is necessary to yield quantitative estimates of underdiagnosis and underreporting of pertussis. We do, however, conclude that the epidemiology of pertussis is largely driven by circulation of the pathogen in adolescent and adult population groups. Other studies have shown that these groups suffer less frequently from symptomatic infection than young children [37]. Therefore, in our efforts to prevent serious infection in young infants, we should take into account that large reservoirs of infection exist and design ways to protect young infants from that risk.

The results presented here, which have been obtained using novel approaches, add to our understanding of the complex transmission dynamics of pertussis infection. On the basis of serological data, we provide a picture of the amount of circulation that is still taking place even in highly vaccinated populations. This information can be used to assess the amount of underdiagnosis and underreporting of pertussis infection and disease. In addition, the novel methods outlined here could be also used to evaluate the impact of the programmatic changes that have been implemented in many countries since these serosurveys were undertaken. In particular the introduction of preschool and adolescent pertussis boosters and the acceleration of the primary schedule were introduced to try to directly protect infants from severe disease.

## Supporting Information

Text S1 Detailed methods used for estimation of incidence.
(0.06 MB DOC)

## Abbreviations

BIC: Bayes Information Criterion
ESEN: European Sero Epidemiology Network
PT: pertussis toxin
