# Supplementary material for: Incidence and Reproduction Numbers of Pertussis: Estimates from Serological and Social Contact Data in Five European Countries
Source: PLoS Med. 2010 Jun 22;7(6):e1000291. doi: 10.1371/journal.pmed.1000291 (PMC2889930; doi:10.1371/journal.pmed.1000291)
Supplement: Text S1 — Detailed methods used for estimation of incidence. (0.06 MB DOC) [file pmed.1000291.s001.doc]

**Supplementary Information with**

Estimating incidence and reproduction numbers of pertussis using serological and social contact data from five European countries

**Mirjam Kretzschmar, Peter Teunis, and Richard Pebody**

**Longitudinal model of the serum antibody response**

The serum antibody response to infection was studied in a data set consisting of repeated samples of IgG-PT titres in 121 patients followed up to 11 years post infection (Versteegh et al 2005). The longitudinal model assumes predator-prey type interaction between antibodies and pathogens (see appendix in Versteegh et al, 2005). Pathogens grow exponentially, presentation of antigen to the immune system is proportional to the numbers of pathogens present. In response the immune system produces antibodies with a rate proportional to the amount of circulating antigen. The rate of inactivation and/or removal of pathogens is assumed proportional to the concentration of circulating antibodies. Turnover of antibodies has first order kinetics with a single fixed rate parameter. This single compartment model is adequate for describing the serum antibody response to infection. Note that antibody levels are here treated as a nuisance variable since we only have observations of serum antibody titres. Antibody responses vary substantially between individual patients (Teunis et al 2002). Therefore the longitudinal model is fitted in a hierarchical framework, allowing the host parameters (antigen dependent antibody production rate and antibody decay rate) to vary among individual patients, while the pathogen parameters (antibody dependent inactivation rate and pathogen growth rate) are assumed the same in all subjects. This approach produces distributions of peak titres and decay rates for IgG-PT antibodies describing the natural variation in serum antibody responses following infection (seroconversion).

**Distribution of serum antibody titres in a cross-sectional population sample**

Estimation of incidence rates was based on a description of the distribution of titres in a cross-sectional sample, in terms of the longitudinal response and the rate with which seroconversions occur. To this end the following simplifying assumptions were made: infections (seroconversions) occur as a time homogeneous Poisson process: the time *t* since the last seroconversion is exponentially distributed at any point in time with density

$$f\left(\left.t\right|\gamma\right)=\gamma\text{e}^{-\gamma t}$$

and rate parameter γ. Seroconversion causes an instantaneous increase in antibody titre *y*(*t*), followed by exponential decay towards baseline.

$$y\left(t\right)=a\text{e}^{-\alpha t}$$

with peak titre *a* and decay rate α. The corresponding distribution of titres in a cross-sectional sample is

$h\left(\left.y\right|\gamma,a,\alpha\right)=\frac{\gamma}{a\alpha}\left(\frac{y}{a}\right)^{\frac{\gamma}{\alpha}-1}$ when 0 ≤ y ≤ a, and 0 elsewhere.

Heterogeneity in serum antibody responses is modelled by assuming distributions for both the peak titre *a* and the decay rate α, as inferred from the longitudinal study, and numerically obtaining the marginal distribution of cross-sectional titres, with the seroconversion rate as a parameter. Thus, the seroconversion rate may be estimated by fitting this marginal distribution to population samples of IgG-PT titres.

**Estimation of incidence based on next generation matrix**

The data is given in the form {(x,a)_n_: n=1,...,N}, where N is the sample size, x denotes the titre value and a the age of the respondent. For classifying individuals as seronegative or seropositive, respectively, we used the cumulative density function

$P(x)=\int_{0}^{x} \Gamma(s,\alpha,\beta)ds$, (S1)

where Γ(s,α,β) denotes the probability density of a Gamma distribution with shape parameter α and scale parameter β. The values for α and β were chosen such (α = 7.3 and β = 11.1) that at a cutoff value of 94 U/ml the sensitivity and specificity agree with values found for diagnostic testing (Baughman et al. 2004). P(x) is then the probability that an individual with IgG PT titre x is classified as seropositive and 1-P(x) the probability that an individual is seronegative. Classifying all N individuals in that way, categorizing into 15 age classes, and reordering leads to the data set {ŷ_k_(i): ŷ_k_(i)=0 for n=1,...,K_i_; ŷ_k_(i)=1 for k=K_i_+1, ..., N_i_; i=1,...,15}. Here N_i_ is the number of respondents in age class i and N_1_+...+N_15_=N. The age classes are 5 year age bands with the exception of the upper age class which contains all data points with ages equal to or greater than 75 years. We can compute the fraction of susceptibles in age class i as the sum of all ŷ_k_(i) in age class i divided by the size of that age class:

for i=1,..., 15.

Now let S(a) denote the fraction of sero-negative persons by age, and λ(a) the age dependent rate of sero-conversion. Furthermore, we denote by 1/γ the time that an individual remains sero-positive after infection. We use IgG PT titre values as a proxy for recent infection rather than a correlate of protection. Then the age dependent fraction of sero-negatives can be described by the differential equation

$\frac{dS}{da}=-\lambda(a)S(a)+\gamma(1-S(a))$ (S2)

with initial value S(0)=1. This equation can be solved explicitly and results in a function describing the fraction of seronegatives by age. For a constant force of infection λ we get

$S(a)=\frac{\gamma+\lambda\exp((-\lambda-\gamma)a)}{\gamma+\lambda}$ (S3)

Assuming that λ is constant in a 5 year age class with upper and lower age bounds a_1_ and a_5_, we can write

$S_{i}=\frac{1}{5}\left(\sum_{k=1}^{5} \frac{\gamma+\lambda_{i}\exp((-\lambda_{i}-\gamma)(a_{k}+0.5))}{\gamma+\lambda_{i}}\right)$ (S4)

i.e. we take the average of the fraction of susceptibles over 5 one year age classes.

The value of the parameter 1/γ was estimated at 1.1 years based on earlier estimates for the decay of antibody titres after infection. Combining these decay rates with the probability function in equ. (S1) led to an estimate for the distribution of the time that an individual will be diagnosed as seropositive after infection. Next, the force of infection λ=( λ_i_), i=1,...,15, was estimated with a maximum likelihood procedure that included an iterative process of determining the next generation matrix M. The next generation matrix M was assumed to be proportional with factor q to an underlying contact matrix C, i.e. M=qC.

$\begin{matrix} \lambda^{n}=qe^{T}Cdiag(1-S_{i}) \\ 1-S_{i}=\lambda_{i}^{n-1}S_{i} \end{matrix}$ (S5)

for i=1,...,15. Here e^T^ is the transposed unit vector and n an index denoting the iteration step. Starting with an arbitrary initial vector λ, the S_i_ can be computed based on (S4). From that estimate a new value of λ can be determined, and so forth. The fixed point of (S5) defines the S_i_. Then the log-likelihood function for *q* can be computed as

(S6)

The log-likelihood function *L(q)* is minimized to find an estimate for *q*. With this estimate the next generation matrix M is computed. The uncertainty in the infectivity parameter q was assessed by using the likelihood function in an iterative adaptive rejection (MCMC) procedure, to obtain a Monte Carlo sample of its distribution. That MC sample was then used to calculate 95% intervals for the incidence and reproduction numbers. Here we did not, however, take uncertainty in the contact matrix C and in γ into account.

As input for the contact matrices C we used the symmetrized mixing matrices from the Polymod surveys for five countries (FI, DE, IT, NL, UK). We did the analysis for the matrices based on all contacts and the matrices based on only those contacts that included physical contact. Furthermore we used two hypothetical contact matrices to further assess the impact of the matrix structure on the estimates for incidence and R_0_. We used a matrix with all elements identical (homogeneous mixing), and a matrix where the diagonal elements of the POLYMOD all contacts matrix was reduced by a multiplication factor 0.2 and the subdiagonals reduced by a factor 0.5. We compared the goodness of fit for all matrices using the Bayes Information Criterion (BIC). Estimates for the basic reproduction number were computed as dominant eigenvalues of the next generation matrix M. We conducted sensitivity analyses to study the impact of the assumptions about α, β, and γ on the estimates for R_0_ and the force of infection (results not shown). Incidence per annum (t=1) was then computed from the estimated fractions of seronegatives per age group and the force of infection by age as

$I(a)=S(a)(1-\exp(-\lambda(a)t))$ (S6)

for one year age groups.

**References**

Teunis PFM, Van Der Heijden OG, De Melker HE, Schellekens JFP, F. Versteegh GA, Kretzschmar MEE (2002). Kinetics of the IgG antibody response to pertussis toxin after infection with *B. Pertussis*. Epidemiol Infect 129(3):479-489

Versteegh FGA, Mertens PLJM, de Melker HE, Roord JJ, Schellekens JFP, Teunis PFM (2005). Age-specific long-term course of IgG antibodies to pertussis toxin after symptomatic infection with *Bordetella pertussis*. Epidemiol Infect 133(4):737-748

Baughman AL, Bisgard KM, Edwards KM, Guris D, Decker MD, et al. (2004) Establishment of diagnostic cutoff points for levels of serum antibodies to pertussis toxin, filamentous hemagglutinin, and fimbriae in adolescents and adults in the United States. Clin Diagn Lab Immunol 11: 1045-1053.
